# Crystal structure of 1,4,8,11-tetra­methyl-1,4,8,11-tetra­azonia­cyclo­tetra­decane bis­[chlorido­chromate(VI)] dichloride from synchrotron X-ray data

**DOI:** 10.1107/S2056989020003059

**Published:** 2020-03-10

**Authors:** Dohyun Moon, Jong-Ha Choi

**Affiliations:** a Beamline Department, Pohang Accelerator Laboratory, POSTECH, Pohang 37673, Republic of Korea; b Department of Chemistry, Andong National University, Andong 36729, Republic of Korea

**Keywords:** crystal structure, 1,4,8,11-tetra­methyl-1,4,8,11-tetra­azonia­cyclo­tetra­decane bis­[chloro­chromate(VI)] dichloride, hydrogen bonding, synchrotron radiation

## Abstract

The crystal structure of title compound, (C_14_H_36_N_4_)[CrO_3_Cl]_2_Cl_2_, has been determined by synchrotron radiation X-ray crystallography at 220 K. The macrocyclic cation lies across a crystallographic inversion center and hence the asymmetric unit contains one half of the organic cation, one chloro­chromate anion and one chloride anion. Both the Cl^−^ anion and chloro­chromate Cl atom are involved in hydrogen bonding. In the crystal, hydrogen bonds involving the 1,4,8,11-tetra­methyl-1,4,8,11-tetra­azonia­cyclo­tetra­decane (TMC) N—H groups and C—H groups as donor groups and three O atoms of the chloro­chromate and the chloride anion as acceptor groups link the components, giving rise to a three-dimensional network.

## Abstract 2

The asymmetric unit of the title compound, (C_14_H_36_N_4_)[CrO_3_Cl]_2_Cl_2_, contains one half-cation (completed by crystallographic inversion symmetry), one chloro­chromate anion and one chloride anion. In the crystal, N—H⋯Cl, C—H⋯Cl and C—H⋯O hydrogen bonds connect the organic cations, chloro­chromate and chloride anions, forming a three-dimensional network.

## Chemical context   

Chromium(VI) compounds have a toxic and genotoxic character to humans and wildlife (Yusof & Malek, 2009 ▸), but they are very important in industrial processes (Goyal *et al.*, 2003 ▸). 1,4,8,11-Tetra­aza­cyclo­tetra­decane and its substituted derivatives are involved in diverse application fields such as catalysis, enzyme mimics, chemical sensors, selective metal-ion recovery, pharmacology and therapy (Meyer *et al.*, 1998 ▸). Tetra-*N*-methyl­ated 1,4,8,11-tetra­methyl-1,4,8,11-tetra­aza­cyclo­tetra­decane (TMC, C_14_H_36_N_4_) is basic and readily captures protons to form a dication, C_14_H_34_N_4_^2+^, or tetra­cation, C_14_H_36_N_4_^4+^, in which the N—H bonds are generally active in hydrogen-bond formation. These organic cations may be suitable for use in the removal of toxic metal ions.

Previously, the crystal structures of [H_4_TMC](ClO_4_)_2_Cl_2_ (Moon & Choi, 2020 ▸), [H_2_TMC][As_4_O_2_Cl_10_], [H_2_TMC][Sb_2_OCl_6_] (Willey *et al.*, 1993 ▸), [H_4_TMC]_2_[Sb_4_F_15_][HF_2_]F_4_ (Becker & Mattes, 1996 ▸), [H_4_TMC][H_2_TMC][W(CN)_8_]_2_·4H_2_O (Nowicka *et al.*, 2012 ▸) and [Al(CH_3_)]_4_[TMC] (Robinson *et al.*, 1987 ▸) were determined, but there is no report of a compound with any combination of the 1,4,8,11-tetra­methyl-1,4,8,11-tetra­azonia­cyclo­tetra­decane cation and CrO_3_Cl^−^ anion. In this communication, we report on the preparation of a new organic chloro­chromate [H_4_TMC][CrO_3_Cl]_2_Cl_2_, (I), and its structural characterization by synchrotron single-crystal X-ray diffraction.
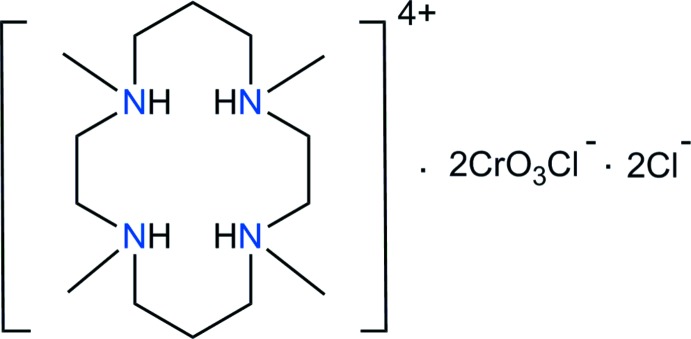


## Structural commentary   

**Figure 1:**
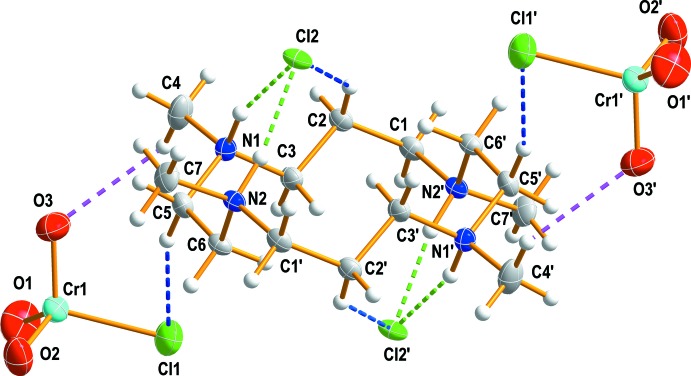
The structure of (I), drawn with displacement ellipsoids at the 50% probability level. Dashed lines represent hydrogen bonding inter­actions and primed atoms are related by the symmetry operation (−*x* + 1, −*y* + 1, −*z*).

**Figure 2:**
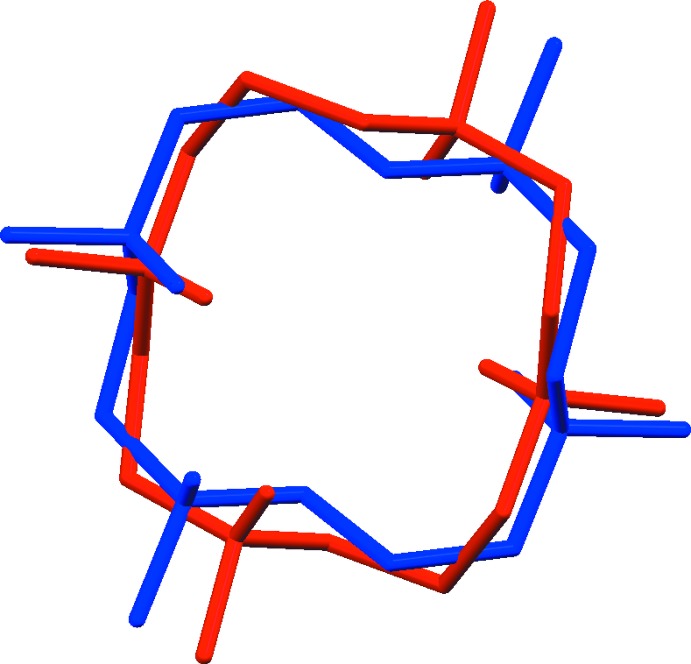
Overlay of the two macrocyclic cations in (I) (blue) and in [H_4_TMC](ClO_4_)_2_Cl_2_ (red).

The mol­ecular structure of (I) is shown in Fig. 1 ▸ along with the atom-numbering scheme. The organic cation lies across a crystallographic inversion center and hence the asymmetric unit contains one half of the organic cation, one chloro­chromate(VI) anion and one chloride anion. The conformation of the tetra­cation in (I) (blue) is similar to that observed in [H_4_TMC](ClO_4_)_2_Cl_2_ (red) (Fig. 2 ▸; r.m.s. deviation overlay = 0.5878 Å), but it is different from the *trans*-I and *trans*-III conformations of the dications in [H_2_TMC][As_4_O_2_Cl_10_] and [H_2_TMC][Sb_2_OCl_6_], respectively (Willey *et al.*, 1993 ▸). Within the centrosymmetric cation unit C_14_H_36_N_4_^4+^, the C—C and N—C bond lengths vary from 1.520 (2) to 1.524 (2) Å and from 1.501 (2) to 1.513 (2) Å, respectively. The ranges of the N—C—C and C—N—C angles are 111.86 (15) to 116.39 (14)° and 108.81 (14) to 112.58 (14)°, respectively. The four nitro­gen atoms of the macrocyclic cation are coplanar with the four nitro­gens occupying the four corners of it with distances between each two N atoms of 3.2242 (13) Å (N1—N2), 5.414 (2) Å (N1—N1′) and 5.5907 (17) Å (N2—N2′), where the primed atoms are related by the symmetry operation (−*x* + 1, −*y* + 1, −*z*). The bond lengths and angles within the tetra­ammonium organic cation are comparable to the corresponding values determined for the H_2_TMC or H_4_TMC moiety in [H_2_TMC][As_4_O_2_Cl_10_], [H_2_TMC][Sb_2_OCl_6_] (Willey *et al.*, 1993 ▸), [H_4_TMC](ClO_4_)_2_Cl_2_ (Moon & Choi, 2020 ▸), [H_4_TMC][H_2_TMC][W(CN)_8_]_2_·4H_2_O (Nowicka *et al.*, 2012 ▸) and [H_4_TMC]_2_[Sb_4_F_15_][HF_2_]F_4_ (Becker & Mattes, 1996 ▸). The CrO_3_Cl^−^ anion exhibits a more or less distorted tetra­hedral geometry (Lorenzo Luis *et al.*, 1996 ▸). The O—Cr—O angles range from 110.49 (14) to 111.22 (13)° and the O—Cr—Cl angles from 108.34 (8) to 109.69 (10)°. The Cr—O bond distances range from 1.588 (2) to 1.602 (2) Å and Cr—Cl bond length is 2.200 (1) Å, in good agreement with the values (2.197 and 2.194 Å) reported for Cs[CrO_3_Cl] and Rb[CrO_3_Cl] (Foster & Sterns, 1974 ▸).

## Supra­molecular features   

**Figure 3:**
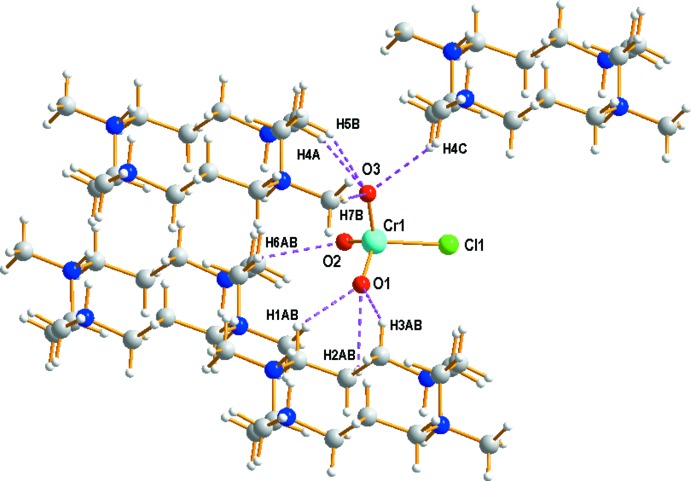
The C—H⋯O hydrogen-bonding inter­actions between neighbouring organic cations and the CrO_3_Cl^−^ anion (see Table 1 ▸ for details).

**Figure 4:**
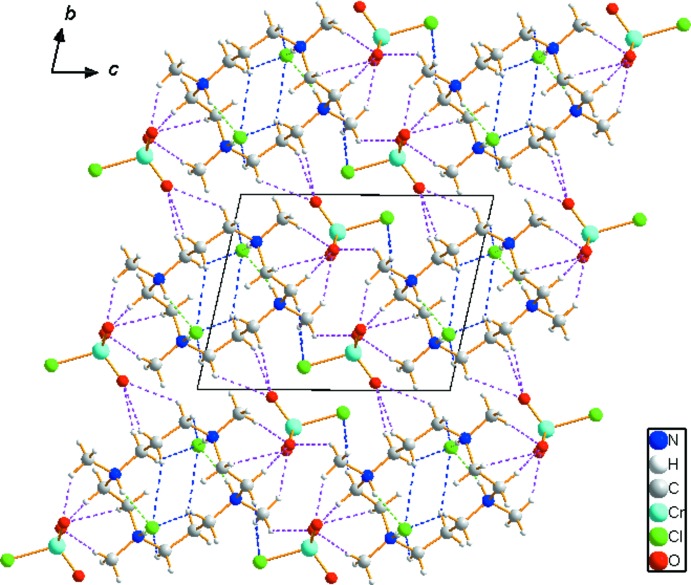
The crystal packing of (I), viewed perpendicular to the *bc* plane. Dashed lines represent N—H⋯Cl (green), C—H⋯O (pink) and C—H⋯Cl (blue) hydrogen-bonding inter­actions (see Table 1 ▸ for details).

Extensive C—H⋯O, C—H⋯Cl and N—H⋯Cl hydrogen-bonding inter­actions occur in the crystal structure (Table 1 ▸). The organic C_14_H_36_N_4_^4+^ cation is linked to two Cl^−^ anions and one CrO_3_Cl^−^ anion *via* N1—H1⋯Cl2, N2—H2⋯Cl2 and C4—H4*C*⋯O3 hydrogen bonds, respectively. In addition, three neighbouring organic cations are inter­connected to the CrO_3_Cl^−^ anion *via* several C—H⋯O hydrogen bonds (Fig. 3 ▸). The extensive array of these contacts generates a three-dimensional network and help to consolidate the crystal structure. The crystal packing diagram of (I) viewed perpendicular to the *bc* plane is shown in Fig. 4 ▸.

## Database survey   

A search of the Cambridge Structural Database (Version 5.41, November 2019; Groom *et al.*, 2016 ▸) indicated only seven hits for organic compounds containing C_14_H_32_N_4_, C_14_H_34_N_4_^2+^ or C_14_H_36_N_4_^4+^ macrocycles: C_14_H_32_N_4_ (refcode LEPXOT; Willey *et al.*, 1994 ▸), [Ga_2_(C_3_H_7_)_4_(OH)_2_](C_14_H_32_N_4_) (XEGGUL; Boag *et al.*, 2000 ▸), Mg_3_Al_13_P_16_O_64_·1.5(C_14_H_32_N_4_)·2.5H_2_O (DAWQUN; Patinec *et al.*, 1999 ▸), [C_14_H_36_N_4_]_2_[Sb_4_F_15_][HF_2_]F_4_ (ZITQUO; Becker *et al.*, 1996 ▸), [C_14_H_34_N_4_][As_4_O_2_Cl_10_] (YALNII; Willey *et al.*, 1993 ▸), [C_14_H_34_N_4_][Sb_2_OCl_6_] (YALNEE; Willey *et al.*, 1993 ▸) and [C_14_H_36_N_4_][C_14_H_34_N_4_][W(CN)_8_]_2_·4H_2_O (ACIKUU; Nowicka *et al.*, 2012 ▸). The conformation of the organic C_14_H_36_N_4_^4+^ cation in (I) is comparable to the *trans*-IV, *trans*-I and *trans*-III conformations of the macrocyclic cations in [C_14_H_36_N_4_](ClO_4_)_2_Cl_2_ (GUCVAE; Moon & Choi, 2020 ▸), [C_14_H_34_N_4_][As_4_O_2_Cl_10_] (YALNII), and [C_14_H_34_N_4_][Sb_2_OCl_6_] (YALNEE), respectively. The *trans*-III and *trans*-IV conformations observed in the two crystallographically independent mol­ecules of C_14_H_32_N_4_ were also comparable (Willey *et al.*, 1994 ▸). However, the compound and structure of any double salt of C_14_H_36_N_4_^4+^ with an additional CrClO_3_^−^ anion is not yet known.

## Synthesis and crystallization   

The free macrocycle TMC (98%) and chromium(VI) trioxide (99%) were purchased from Sigma–Aldrich and used without further purification. All other chemicals were reagent-grade materials and used as received. To a solution of TMC (0.128 g, 0.5 mmol) in 6 *M* HCl (15 mL) was added a solution of chromium(VI) trioxide (0.1 g, 1 mmol) in 6 *M* HCl (5 mL) at 298 K. The resulting solution was stirred for 2 h and left to stand for slow evaporation at room temperature. Block-like red single crystals of (I) suitable for X-ray analysis were obtained by filtration.

## Refinement   

Crystal data, data collection and structure refinement details are summarized in Table 2 ▸. All H atoms were placed in geometrically idealized positions and constrained to ride on their parent atoms, with C—H = 0.97–0.98 Å and N—H = 0.99 Å, respectively, and with *U*_iso_(H) values of 1.5 and 1.2*U*_eq_ of the parent atoms.

## Supplementary Material

Crystal structure: contains datablock(s) I. DOI: 10.1107/S2056989020003059/vm2229sup1.cif

Structure factors: contains datablock(s) I. DOI: 10.1107/S2056989020003059/vm2229Isup2.hkl

CCDC reference: 1988052

Additional supporting information:  crystallographic information; 3D view; checkCIF report

## Figures and Tables

**Table 1:** Hydrogen-bond geometry (Å, °)

*D*—H⋯*A*	*D*—H	H⋯*A*	*D*⋯*A*	*D*—H⋯*A*
N1—H1⋯Cl2	0.99	2.16	3.120 (2)	163
N2—H2⋯Cl2	0.99	2.08	3.0553 (17)	170
C2—H2*A*⋯Cl2	0.98	2.85	3.642 (2)	139
C4—H4*C*⋯O3	0.97	2.58	3.299 (4)	131
C5—H5*AB*⋯Cl1	0.98	2.86	3.804 (2)	161
C3—H3*A*⋯Cl2^i^	0.98	2.72	3.541 (2)	142
C1—H1*A*⋯Cl2^ii^	0.98	2.74	3.511 (2)	136
C1—H1*AB*⋯O1^iii^	0.98	2.59	3.230 (3)	123
C2—H2*AB*⋯O1^iii^	0.98	2.54	3.047 (3)	112
C3—H3*AB*⋯O1^iii^	0.98	2.53	3.193 (3)	125
C4—H4*A*⋯O3^iv^	0.97	2.34	3.168 (3)	143
C5—H5*A*⋯O3^iv^	0.98	2.40	3.218 (3)	141
C7—H7*B*⋯O3^iv^	0.97	2.38	3.343 (3)	173
C4—H4*B*⋯Cl1^v^	0.97	2.88	3.773 (3)	154
C6—H6*AB*⋯O2^vi^	0.98	2.54	3.260 (3)	130

Symmetry codes: (i) 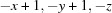; (ii) 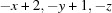; (iii) 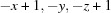; (iv) 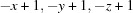; (v) 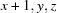; (vi) 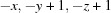.

**Table 2:** Experimental details

Crystal data	
Chemical formula	(C_14_H_36_N_4_)[CrO_3_Cl]_2_Cl_2_
*M*_r_	602.27
Crystal system, space group	Triclinic, *P* 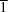
Temperature (K)	220
*a*, *b*, *c* (Å)	7.0610 (14), 8.6740 (17), 10.775 (2)
α, β, γ (°)	77.61 (3), 88.20 (3), 79.39 (3)
*V* (Å^3^)	633.5 (2)
*Z*	1
Radiation type	Synchrotron, λ = 0.610 Å
μ (mm^−1^)	0.85
Crystal size (mm)	0.21 × 0.15 × 0.11

Data collection	
Diffractometer	Rayonix MX225HS CCD area detector
Absorption correction	Empirical (using intensity measurements) (*HKL3000sm *SCALEPACK**; Otwinowski & Minor, 1997 ▸)
*T*_min_, *T*_max_	0.552, 1.000
No. of measured, independent and observed [*I* > 2σ(*I*)] reflections	6933, 3503, 3357
*R*_int_	0.022
(sin θ/λ)_max_ (Å^−1^)	0.693

Refinement	
*R*[*F* ^2^ > 2σ(*F* ^2^)], *wR*(*F* ^2^), *S*	0.050, 0.150, 1.08
No. of reflections	3503
No. of parameters	138
H-atom treatment	H-atom parameters constrained
Δρ_max_, Δρ_min_ (e Å^−3^)	0.81, −1.09

Computer programs: *PAL BL2D-SMDC* (Shin *et al.*, 2016 ▸), *HKL3000sm* (Otwinowski & Minor, 1997 ▸), *SHELXT2018* (Sheldrick, 2015*a* ▸), *SHELXL2018* (Sheldrick, 2015*b* ▸), *DIAMOND4* (Putz & Brandenburg, 2014 ▸) and *publCIF* (Westrip, 2010 ▸).

## supplementary crystallographic information

### Crystal data

**Table:**

(C_14_H_36_N_4_)[CrO_3_Cl]_2_Cl_2_	*Z* = 1
*M**_r_* = 602.27	*F*(000) = 312
Triclinic, *P*1	*D*_x_ = 1.579 Mg m^−^^3^
*a* = 7.0610 (14) Å	Synchrotron radiation, λ = 0.610 Å
*b* = 8.6740 (17) Å	Cell parameters from 41622 reflections
*c* = 10.775 (2) Å	θ = 0.4–33.7°
α = 77.61 (3)°	µ = 0.85 mm^−^^1^
β = 88.20 (3)°	*T* = 220 K
γ = 79.39 (3)°	Block, red
*V* = 633.5 (2) Å^3^	0.21 × 0.15 × 0.11 mm

### Data collection

**Table:**

Rayonix MX225HS CCD area detector diffractometer	3357 reflections with *I* > 2σ(*I*)
Radiation source: PLSII 2D bending magnet	*R*_int_ = 0.022
ω scan	θ_max_ = 25.0°, θ_min_ = 1.7°
Absorption correction: empirical (using intensity measurements) (*HKL3000sm Scalepack*; Otwinowski & Minor, 1997)	*h* = −9→9
*T*_min_ = 0.552, *T*_max_ = 1.000	*k* = −12→12
6933 measured reflections	*l* = −14→14
3503 independent reflections	

### Refinement

**Table:**

Refinement on *F*^2^	0 restraints
Least-squares matrix: full	Hydrogen site location: inferred from neighbouring sites
*R*[*F*^2^ > 2σ(*F*^2^)] = 0.050	H-atom parameters constrained
*wR*(*F*^2^) = 0.150	*w* = 1/[σ^2^(*F*_o_^2^) + (0.1021*P*)^2^ + 0.4428*P*] where *P* = (*F*_o_^2^ + 2*F*_c_^2^)/3
*S* = 1.08	(Δ/σ)_max_ < 0.001
3503 reflections	Δρ_max_ = 0.81 e Å^−^^3^
138 parameters	Δρ_min_ = −1.08 e Å^−^^3^

### Special details

**Table:**

Geometry. All esds (except the esd in the dihedral angle between two l.s. planes) are estimated using the full covariance matrix. The cell esds are taken into account individually in the estimation of esds in distances, angles and torsion angles; correlations between esds in cell parameters are only used when they are defined by crystal symmetry. An approximate (isotropic) treatment of cell esds is used for estimating esds involving l.s. planes.

### Fractional atomic coordinates and isotropic or equivalent isotropic displacement parameters (Å^2^)

**Table:**

	*x*	*y*	*z*	*U*_iso_*/*U*_eq_	
N1	0.5951 (2)	0.43908 (19)	0.24944 (14)	0.0197 (3)	
H1	0.655924	0.528759	0.203398	0.024*	
N2	0.3111 (2)	0.75634 (17)	0.10259 (15)	0.0178 (3)	
H2	0.448197	0.736710	0.077822	0.021*	
C1	0.8079 (3)	0.1708 (2)	0.01696 (18)	0.0203 (3)	
H1A	0.942223	0.140900	−0.007767	0.024*	
H1AB	0.763915	0.071671	0.058488	0.024*	
C2	0.8030 (2)	0.2755 (2)	0.11389 (17)	0.0202 (3)	
H2A	0.850838	0.373545	0.074404	0.024*	
H2AB	0.889395	0.217967	0.184918	0.024*	
C3	0.6013 (3)	0.3211 (2)	0.16488 (18)	0.0216 (3)	
H3A	0.511705	0.368064	0.093415	0.026*	
H3AB	0.559012	0.224125	0.212911	0.026*	
C4	0.7083 (4)	0.3635 (3)	0.3696 (2)	0.0350 (5)	
H4A	0.710246	0.443738	0.419628	0.052*	
H4B	0.839127	0.320257	0.348638	0.052*	
H4C	0.648561	0.277582	0.418098	0.052*	
C5	0.3917 (3)	0.5084 (2)	0.28166 (17)	0.0214 (3)	
H5A	0.398383	0.581772	0.338417	0.026*	
H5AB	0.331618	0.420559	0.329015	0.026*	
C6	0.2612 (2)	0.5985 (2)	0.16973 (17)	0.0191 (3)	
H6A	0.265642	0.529620	0.108100	0.023*	
H6AB	0.128516	0.617792	0.200171	0.023*	
C7	0.2870 (3)	0.8760 (2)	0.1871 (2)	0.0287 (4)	
H7A	0.309296	0.978749	0.138299	0.043*	
H7B	0.378911	0.838265	0.256843	0.043*	
H7C	0.157143	0.888100	0.220467	0.043*	
Cr1	0.20005 (5)	0.19152 (4)	0.58525 (3)	0.02581 (14)	
Cl1	0.21038 (11)	0.11680 (9)	0.40216 (7)	0.04726 (19)	
O1	0.2178 (4)	0.0382 (3)	0.6983 (2)	0.0578 (6)	
O2	0.0005 (3)	0.3102 (3)	0.59266 (19)	0.0461 (5)	
O3	0.3790 (3)	0.2812 (3)	0.5855 (2)	0.0459 (5)	
Cl2	0.74460 (6)	0.71108 (6)	0.05475 (5)	0.02599 (15)	

### Atomic displacement parameters (Å^2^)

**Table:**

	*U*^11^	*U*^22^	*U*^33^	*U*^12^	*U*^13^	*U*^23^
N1	0.0203 (7)	0.0201 (7)	0.0179 (6)	−0.0016 (5)	−0.0003 (5)	−0.0042 (5)
N2	0.0161 (6)	0.0139 (6)	0.0231 (7)	−0.0019 (5)	0.0036 (5)	−0.0049 (5)
C1	0.0175 (7)	0.0155 (7)	0.0252 (8)	0.0018 (6)	0.0016 (6)	−0.0024 (6)
C2	0.0168 (7)	0.0194 (8)	0.0226 (8)	−0.0002 (6)	0.0009 (6)	−0.0030 (6)
C3	0.0189 (8)	0.0223 (8)	0.0244 (8)	−0.0023 (6)	0.0029 (6)	−0.0085 (6)
C4	0.0384 (12)	0.0407 (12)	0.0216 (9)	0.0067 (9)	−0.0102 (8)	−0.0078 (8)
C5	0.0220 (8)	0.0216 (8)	0.0192 (7)	−0.0018 (6)	0.0050 (6)	−0.0038 (6)
C6	0.0174 (7)	0.0155 (7)	0.0239 (8)	−0.0034 (5)	0.0041 (6)	−0.0030 (6)
C7	0.0381 (11)	0.0196 (8)	0.0312 (9)	−0.0048 (7)	0.0047 (8)	−0.0124 (7)
Cr1	0.0292 (2)	0.0227 (2)	0.0242 (2)	−0.00618 (13)	0.00070 (13)	−0.00076 (13)
Cl1	0.0520 (4)	0.0530 (4)	0.0450 (4)	−0.0119 (3)	0.0076 (3)	−0.0274 (3)
O1	0.0855 (18)	0.0349 (10)	0.0450 (11)	−0.0130 (10)	−0.0045 (11)	0.0111 (8)
O2	0.0433 (10)	0.0534 (11)	0.0367 (9)	0.0066 (9)	0.0027 (7)	−0.0126 (8)
O3	0.0468 (11)	0.0535 (12)	0.0454 (10)	−0.0246 (9)	−0.0010 (8)	−0.0141 (9)
Cl2	0.0172 (2)	0.0255 (2)	0.0356 (3)	−0.00824 (16)	0.00510 (17)	−0.00431 (19)

### Geometric parameters (Å, º)

**Table:**

N1—C4	1.501 (2)	C4—H4A	0.9700
N1—C3	1.503 (2)	C4—H4B	0.9700
N1—C5	1.511 (2)	C4—H4C	0.9700
N1—H1	0.9900	C5—C6	1.521 (3)
N2—C6	1.503 (2)	C5—H5A	0.9800
N2—C7	1.505 (2)	C5—H5AB	0.9800
N2—C1^i^	1.513 (2)	C6—H6A	0.9800
N2—H2	0.9900	C6—H6AB	0.9800
C1—C2	1.520 (3)	C7—H7A	0.9700
C1—H1A	0.9800	C7—H7B	0.9700
C1—H1AB	0.9800	C7—H7C	0.9700
C2—C3	1.524 (2)	Cr1—O1	1.588 (2)
C2—H2A	0.9800	Cr1—O2	1.596 (2)
C2—H2AB	0.9800	Cr1—O3	1.602 (2)
C3—H3A	0.9800	Cr1—Cl1	2.2000 (9)
C3—H3AB	0.9800		
			
C4—N1—C3	110.93 (15)	N1—C4—H4A	109.5
C4—N1—C5	109.63 (15)	N1—C4—H4B	109.5
C3—N1—C5	112.58 (14)	H4A—C4—H4B	109.5
C4—N1—H1	107.8	N1—C4—H4C	109.5
C3—N1—H1	107.8	H4A—C4—H4C	109.5
C5—N1—H1	107.8	H4B—C4—H4C	109.5
C6—N2—C7	112.00 (15)	N1—C5—C6	116.12 (14)
C6—N2—C1^i^	112.21 (14)	N1—C5—H5A	108.3
C7—N2—C1^i^	108.81 (14)	C6—C5—H5A	108.3
C6—N2—H2	107.9	N1—C5—H5AB	108.3
C7—N2—H2	107.9	C6—C5—H5AB	108.3
C1^i^—N2—H2	107.9	H5A—C5—H5AB	107.4
N2^i^—C1—C2	116.39 (14)	N2—C6—C5	115.04 (15)
N2^i^—C1—H1A	108.2	N2—C6—H6A	108.5
C2—C1—H1A	108.2	C5—C6—H6A	108.5
N2^i^—C1—H1AB	108.2	N2—C6—H6AB	108.5
C2—C1—H1AB	108.2	C5—C6—H6AB	108.5
H1A—C1—H1AB	107.3	H6A—C6—H6AB	107.5
C1—C2—C3	112.61 (15)	N2—C7—H7A	109.5
C1—C2—H2A	109.1	N2—C7—H7B	109.5
C3—C2—H2A	109.1	H7A—C7—H7B	109.5
C1—C2—H2AB	109.1	N2—C7—H7C	109.5
C3—C2—H2AB	109.1	H7A—C7—H7C	109.5
H2A—C2—H2AB	107.8	H7B—C7—H7C	109.5
N1—C3—C2	111.86 (15)	O1—Cr1—O2	110.49 (14)
N1—C3—H3A	109.2	O1—Cr1—O3	111.05 (14)
C2—C3—H3A	109.2	O2—Cr1—O3	111.22 (13)
N1—C3—H3AB	109.2	O1—Cr1—Cl1	109.69 (10)
C2—C3—H3AB	109.2	O2—Cr1—Cl1	108.34 (8)
H3A—C3—H3AB	107.9	O3—Cr1—Cl1	105.90 (9)
			
N2^i^—C1—C2—C3	61.3 (2)	C3—N1—C5—C6	−59.5 (2)
C4—N1—C3—C2	−67.1 (2)	C7—N2—C6—C5	−64.42 (19)
C5—N1—C3—C2	169.64 (15)	C1^i^—N2—C6—C5	172.85 (14)
C1—C2—C3—N1	−173.71 (14)	N1—C5—C6—N2	−69.9 (2)
C4—N1—C5—C6	176.52 (17)		

Symmetry code: (i) −*x*+1, −*y*+1, −*z*.

### Hydrogen-bond geometry (Å, º)

**Table:**

*D*—H···*A*	*D*—H	H···*A*	*D*···*A*	*D*—H···*A*
N1—H1···Cl2	0.99	2.16	3.120 (2)	163
N2—H2···Cl2	0.99	2.08	3.0553 (17)	170
C2—H2*A*···Cl2	0.98	2.85	3.642 (2)	139
C4—H4*C*···O3	0.97	2.58	3.299 (4)	131
C5—H5*AB*···Cl1	0.98	2.86	3.804 (2)	161
C3—H3*A*···Cl2^i^	0.98	2.72	3.541 (2)	142
C1—H1*A*···Cl2^ii^	0.98	2.74	3.511 (2)	136
C1—H1*AB*···O1^iii^	0.98	2.59	3.230 (3)	123
C2—H2*AB*···O1^iii^	0.98	2.54	3.047 (3)	112
C3—H3*AB*···O1^iii^	0.98	2.53	3.193 (3)	125
C4—H4*A*···O3^iv^	0.97	2.34	3.168 (3)	143
C5—H5*A*···O3^iv^	0.98	2.40	3.218 (3)	141
C7—H7*B*···O3^iv^	0.97	2.38	3.343 (3)	173
C4—H4*B*···Cl1^v^	0.97	2.88	3.773 (3)	154
C6—H6*AB*···O2^vi^	0.98	2.54	3.260 (3)	130

Symmetry codes: (i) −*x*+1, −*y*+1, −*z*; (ii) −*x*+2, −*y*+1, −*z*; (iii) −*x*+1, −*y*, −*z*+1; (iv) −*x*+1, −*y*+1, −*z*+1; (v) *x*+1, *y*, *z*; (vi) −*x*, −*y*+1, −*z*+1.
